# A needle arthroscopy approach to the temporomandibular joints in dogs

**DOI:** 10.3389/fvets.2026.1760965

**Published:** 2026-01-28

**Authors:** Boaz Arzi, Stephanie Goldschmidt, Emilia Chrostek, Minh Vo Duong, Barbro Filliquist, Po-Yen Chou

**Affiliations:** 1 Department of Surgical and Radiological Sciences, School of Veterinary Medicine, University of California, Davis, Davis, CA, United States; 2 William R. Pritchard Veterinary Teaching Hospital, School of Veterinary Medicine, University of California, Davis, Davis, CA, United States; 3 Department of Veterinary Clinical Sciences, Faculty of Animal Science and Veterinary Medicine, Nong Lam University, Ho Chi Minh City, Vietnam

**Keywords:** diagnostics, minimally invasive, NanoNeedle Scope, osteoarthritis, temporomandibular joint

## Abstract

The temporomandibular joint (TMJ) has been identified as an important contributor to orofacial pain and dysfunction in dogs. Although TMJ disorders have been partially characterized, a more comprehensive understanding and development of diagnostic and therapeutic options are needed. Arthroscopy of the TMJ is a technique that allows for direct visualization and inspection of the internal joint structures, in addition to the collection of biopsies and the performance of select surgical procedures under the operator’s direct visual control. This Methods article, illustrated by two clinical cases, demonstrates the practical and efficient use of needle arthroscopy in dogs’ TMJs. Understanding the fundamental aspects of TMJ arthroscopy is important for diagnosing and treating TMJ conditions, especially given the TMJ’s unique anatomical features in dogs. A semi-open approach was found to be more practical and less likely to cause iatrogenic damage than a single stab incision. The ability to visualize joint structures in health and disease and to perform arthroscopy-guided biopsies is valuable and carries clinically relevant implications.

## Introduction

The temporomandibular joint (TMJ) plays an essential role in the biomechanics of the mammalian jaw, providing essential functions such as chewing, drinking, vocalizing, and swallowing (1, 2). For over a decade, TMJ disorders have been identified as an important cause of orofacial pain and dysfunction in animals (1, 3–11). Among domesticated animals, TMJ disorders in dogs and cats have been characterized by an incidence rate that warrants a comprehensive understanding of the condition and the development of diagnostic and therapeutic options (3, 5, 7, 12, 13).

In both animals and humans, TMJ pathology encompasses a spectrum of conditions, ranging from degenerative joint diseases with associated arthritides to traumatic injuries and neoplasia (14–18). Accurate diagnosis is fundamental to implementing effective treatment strategies and necessitates the utilization of advanced diagnostic methodologies. To date, these have primarily included imaging techniques such as computed tomography (CT), cone beam CT (2) and magnetic resonance imaging (MRI) (7, 11, 19, 20).

Temporomandibular joint arthroscopy is an underutilized technique in veterinary medicine; however, it allows direct visualization and inspection of the internal joint structures. It also enables obtaining biopsies and performing selected surgical procedures under the operator’s direct visual control (21, 22). In humans, TMJ arthroscopy was introduced by Ohinishi et al. in Japan in 1980 and in 1986 in the United States for the diagnosis and treatment of TMJ internal derangement (15, 23). TMJ arthroscopy is now a fairly common procedure in humans and has evolved into a valuable tool for both diagnostic and therapeutic purposes, offering advantages such as reduced morbidity, quicker recovery times, and improved patient outcomes (22, 24–26).

In veterinary medicine, applications of clinical TMJ arthroscopy have primarily been limited to horses due to size constraints (27, 28). However, technological advancements have further enhanced the precision and capabilities of arthroscopy, particularly with the introduction of needle arthroscopy (28–30). The small diameter of the needle arthroscope provides unparalleled visualization of joint structures, particularly in small joints, allowing for a more detailed and accurate assessment of pathological changes (31–33). Recently, an examination protocol was developed and tested on cadaver dog heads using a needle arthroscope (29). The investigators evaluated 15 anatomical landmarks in the TMJs of dogs for visibility and accessibility and demonstrated that TMJ arthroscopy performed with a needle arthroscope can reliably be visualized and assessed with 86–100% certainty (29).

While needle arthroscopy has demonstrated promising results in various joints, its application to the TMJs in dogs has only recently been explored in cadavers (29). Therefore, the purpose of this Methods report was to provide our clinical description of the needle arthroscopy technique in large-breed dogs. We aimed to (1) develop and describe a practical arthroscopic approach to the TMJs of dogs that could be applied in clinical settings; (2) determine the limitations of the TMJ arthroscopic procedure; and (3) exemplify the use of TMJ arthroscopy in two clinical cases.

## Material and equipment

The NanoScope™ arthroscopy system (Arthrex Vet Systems Inc., Naples, FL, United States) was used for the TMJ arthroscopy. The NanoScope console™ was set on a Mayo stand near the patient, outside the sterile field. The NanoScope utilizes a “chip-on-tip” design, integrating a solid-state image sensor and LED light source directly at the distal end of a 1.9 mm semirigid scope. Unlike traditional telescopes, it contains no fiber optics or lenses. This single-use, 0-degree scope provides a 120° field of view and connects to a portable console for image display.

A sterile field was created for the NanoNeedle Scope handpiece kit. The handpiece was connected to the console, and calibration was performed according to the manufacturer’s instructions. Using a sharp obturator, the 2.2 mm inflow sheath was introduced into the joint spaces. Once the cannula was positioned inside the dorsal or ventral joint compartments (see next section), the obturator was removed, and the 125 mm,1.9 mm NanoNeedle Scope was inserted into the sheath and locked into place. A two-way stopcock fluid port was attached to the sheath for fluid management.

Visualization of the articular structure was achieved by injecting sterile saline through the fluid port. Still images and videos were then obtained. At the conclusion of the procedure, the joint was lavaged via the fluid port, and the NanoNeedle Scope with sheath was removed.

## Methods

**Figure 1:**
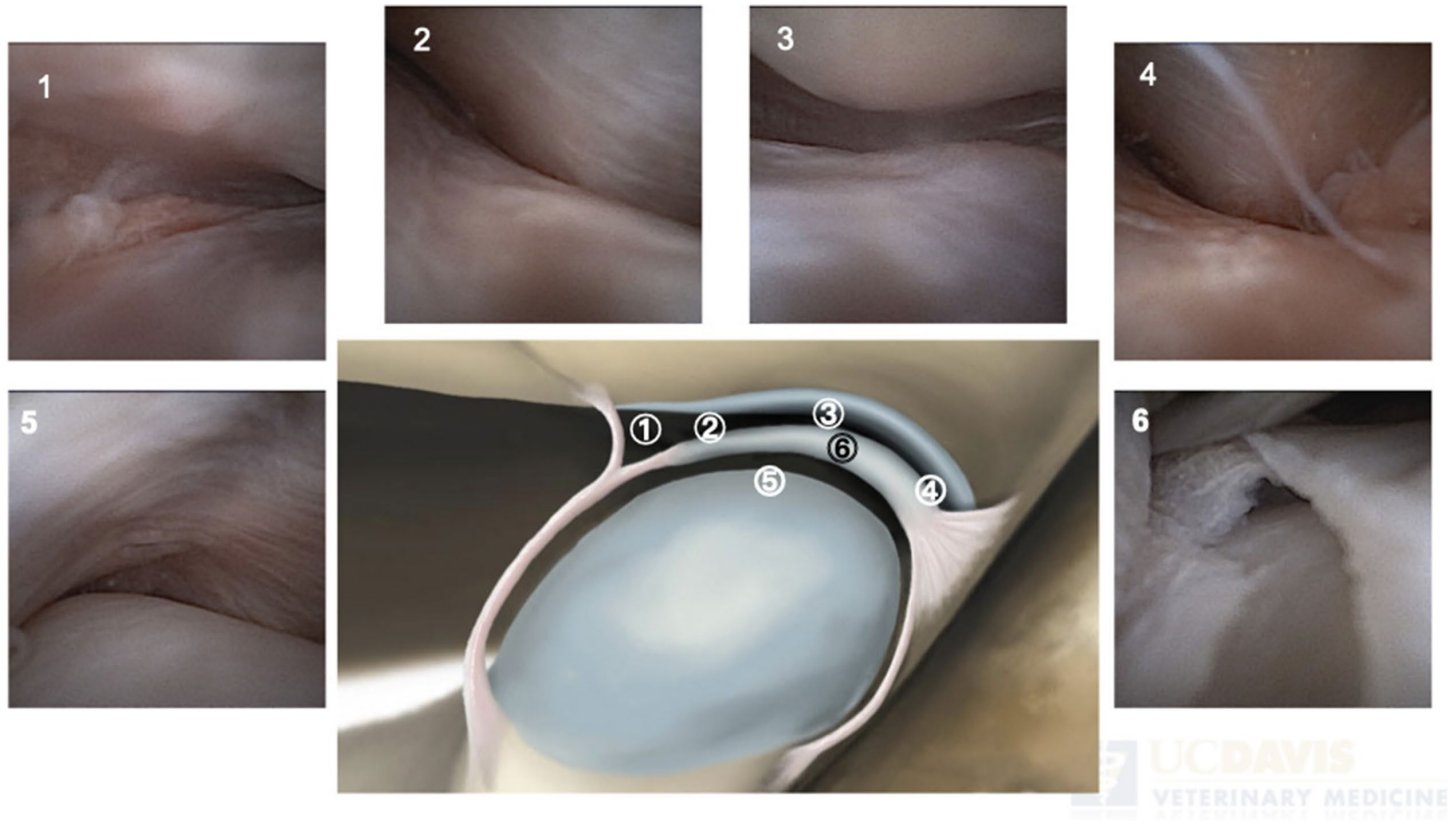
Representative arthroscopic images of a dog’s TMJ using NanoNeedle scope technology. The relevant anatomical landmarks are labeled in the sagittal cross-sectional illustration of the TMJ, with the rostral aspect of the joint on the left. The rostral discotemporal joint with clear visualization of the rostral synovium (1), the synovial discoid junction (disc attachment) and the rostral border of the mandibular fossa (2), the rostral protrusion of the mandibular fossa with the disc (3), the caudal discotemporal joint with visualization of the caudal synovial pouch (4), the discomandibular joint space (5), iatrogenic disc perforation during NanoScope insertion. The mandibular articular surface can be visualized through the disc perforation (6).

- Closed arthroscopic approach: For this part of the study, four large-breed dog cadaver heads (two mesaticephalic and two brachycephalic) were used. All dogs had been humanely euthanized for reasons not related to this study. Following surgical preparation of the TMJ area and with the cadaver head placed in lateral recumbency, a 1 ½ inch, 22-gauge needle was introduced percutaneously into the TMJ. Following verification of needle placement in the temporomandibular joint space by tactile feel (i.e., the sensation of a joint cavity with no resistance), a 10 mm incision was made in the skin and subcutaneous tissue cranially and caudally to the needle using a #15 scalpel blade. Once the location of the dorsal joint space was confirmed, the inflow sheath with obturator was inserted into the TMJ while slightly opening and closing the mouth to assist with the insertion of the sheath. Once in the joint, the obturator was removed, and the NanoNeedle scope was inserted. The joint was flushed with sterile saline to improve visualization. When this initial approach was used on the first eight cadaveric TMJs, linear damage to the TMJ disc was observed in all cases upon arthroscopic examination, likely due to the blind trocar sheath insertion. Therefore, the surgical approach was modified to a semi-open technique, which is described below.
- A semi-open approach to the TMJ was performed with the dog’s head in lateral recumbency. Following surgical preparation, an incision of approximately 30 mm was made over the ventral aspect of the zygomatic arch, at the dorsal aspect of the TMJ, and over the mandibular fossa area. Following blunt and sharp dissection, the joint capsule was incised horizontally. The TMJ disc was then freed from its dorsal and ventral attachments and held with Adson forceps. The dorsal and ventral joint cavities were exposed to facilitate the safe entrance of the inflow sheath and blunt obturator. The first entrance was into the dorsal joint cavity and was performed while gently opening and closing the mouth. The obturator was then removed, and the NanoNeedle Scope was inserted. The center of the joint and the disc were visualized by identifying the gliding motion of the TMJ disc during range of motion testing. The rostral, medial, and caudal articular surfaces of the mandibular fossa were assessed. Once visualization and inspection were concluded, the sheath and the NanoNeedle Scope were completely retracted, and the same process of insertion was performed in the ventral compartment. The articular surface of the mandibular head at the central area and the disc were visualized, followed by an evaluation of the rostral, medial, and caudal articular surfaces and disc attachments (Figure 1).

## Case example

### Case 1

**Figure 2:**
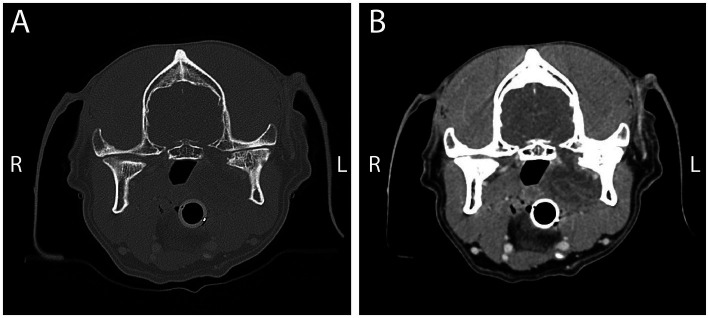
Conventional CT images pre- **(A)** and post- **(B)** contrast at the level of the TMJ in a 12-year-old German shorthaired pointer, as described in Case 1. The left condylar process exhibits bone lysis at the medial aspect of the mandibular head and subchondral bone changes. The contrast study reveals a soft tissue mass, likely arising from the left TMJ at the medial aspect of the joint, and pressing on the pharyngeal area.

A 12-year-old, 39.6 kg, German shorthaired pointer was presented for biopsy of a presumed neoplastic process arising from the left TMJ. The dog was referred due to exophthalmos of the left eye. Conventional CT with and without contrast was performed by the referring veterinarian, and revealed a large hypoattenuating mass at the medial aspect of the left TMJ (Figure 2), extending into the retrobulbar area, causing the exophthalmos. Based on the imaging findings, the primary differential diagnoses were orbital/juxta-articular myxosarcoma, myxoma, or a poorly circumscribed abscess with secondary osteomyelitis. Changes in the mandibular head of the condylar process included an articular surface abrasion, a subchondral cyst, and bone lysis. The majority of the mass was present medial to the left TMJ, posing a biopsy challenge due to the proximity of the maxillary artery and no visual perforation of the mass into the oral cavity.

**Figure 3:**
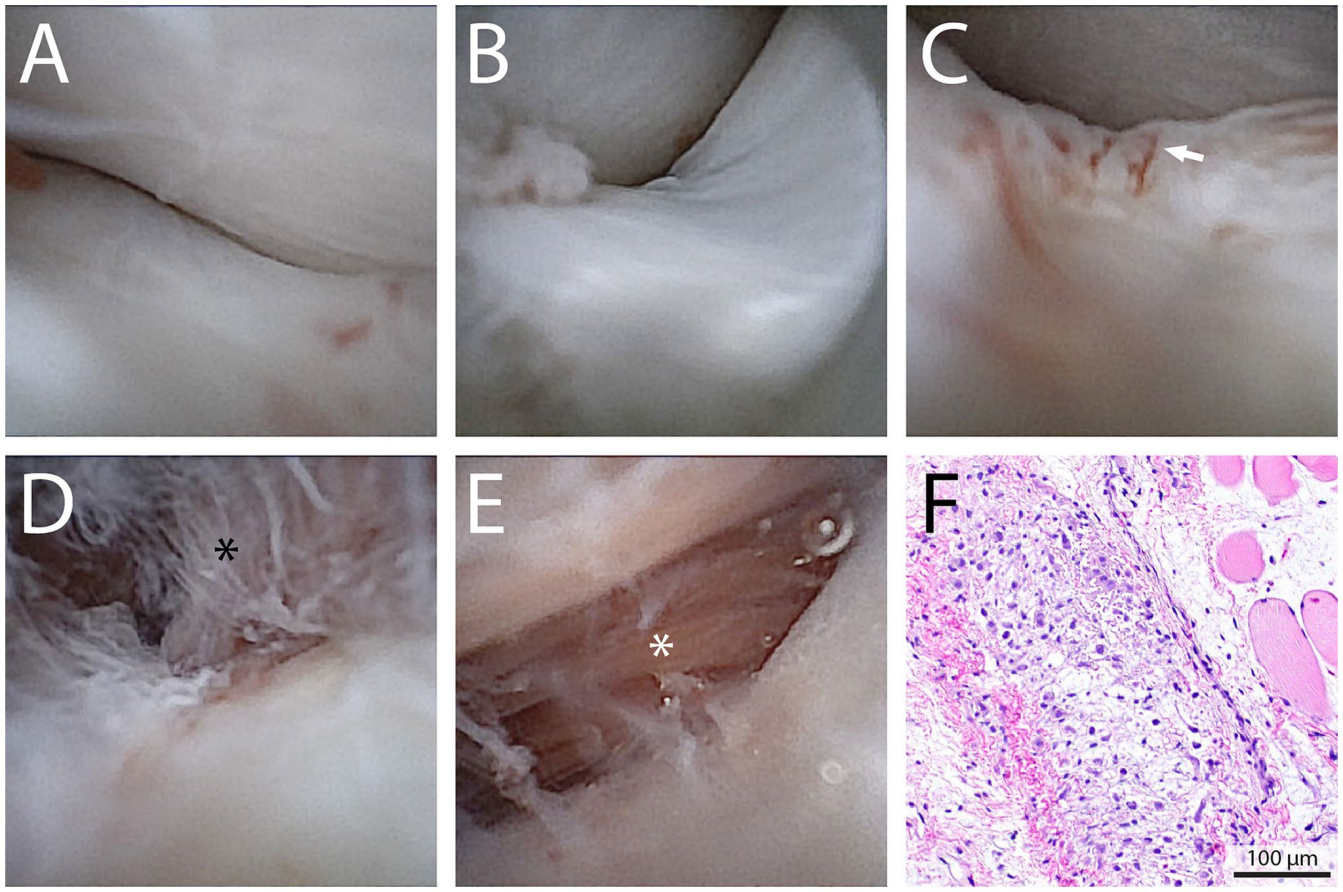
Intra-articular view of the left TMJ of the dog depicted in Case 1. The articular surfaces and the discs at the central **(A)** and caudal **(B)** surfaces, along with the rest of the dorsal compartments, were found within normal limits. The articular surface of the mandibular head, as viewed from the ventral compartment, exhibited erosion of the articular surfaces **(C)**. Fibrous tears were noted in the medial aspect of the joint in the area of disc attachment **(D)**. Finally, brownish-pink abnormal tissue was noted in the media-rostral aspect of the joint **(E)**. Biopsies were obtained, and the clinico-pathologic correlation revealed myxosarcoma **(F)**.

**Figure 4:**
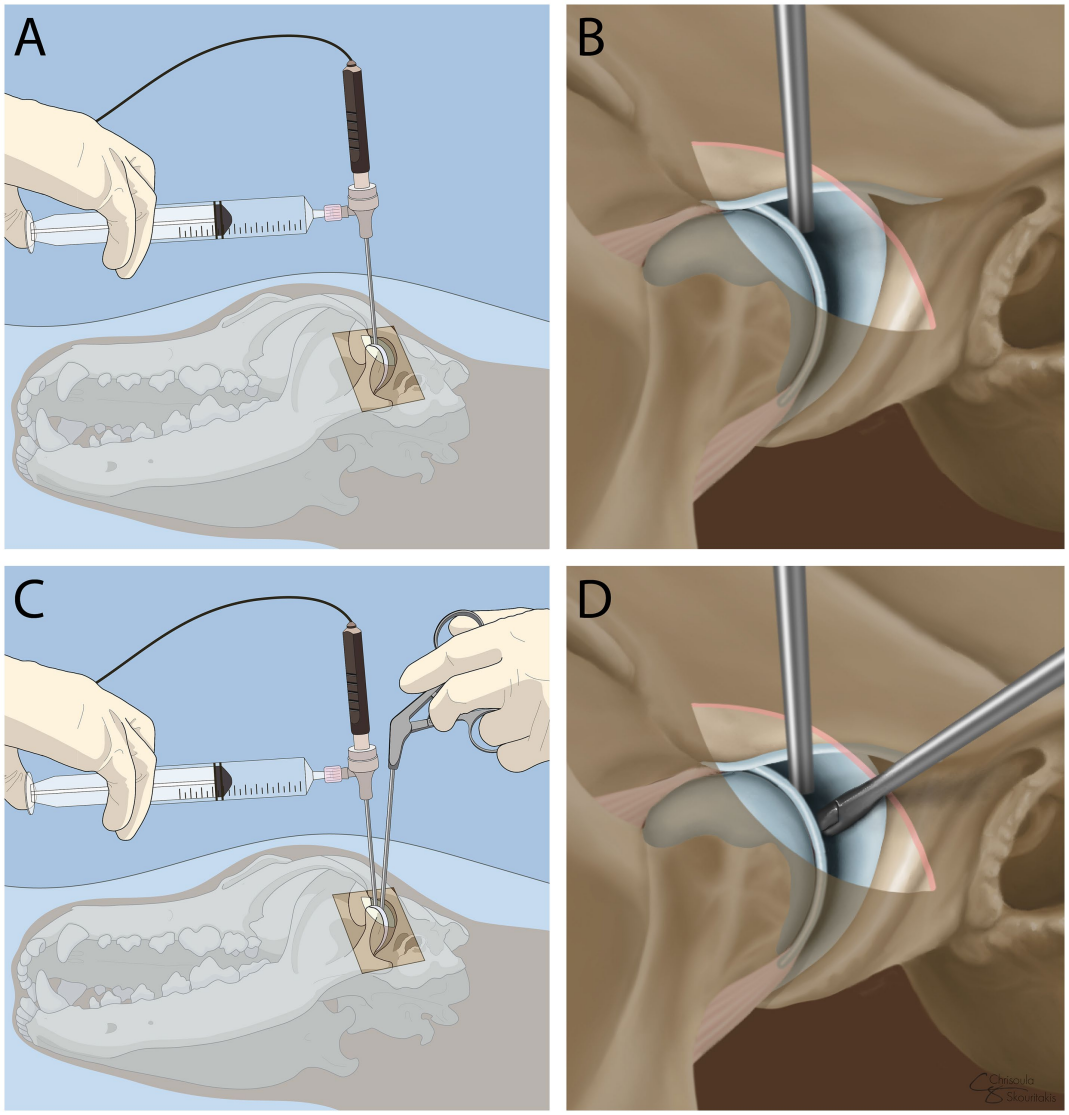
Illustration demonstrating the positioning of the NanoScope in the dorsal joint compartment **(A,B)** and the positioning of the graspers adjacent to the cannula used to obtain biopsies **(C,D)**.

With the dog in lateral recumbency, a lateral approach to the TMJ was performed as previously described, followed by a semi-open arthroscopy. The first approach was to the dorsal compartment of the left TMJ. The articular surfaces of the mandibular fossa and disc at the central, medial, rostral, and caudal regions were inspected and found to be within normal limits (Figures 3A,B). The sheath was then removed and inserted into the ventral compartment, as previously described. Upon entering the ventral compartment, severe erosion of the articular surfaces was noted, including the entire mandibular head (Figure 3C). In addition, fibrous tears were noted in the medial aspect of the joint, in the area of disc attachment (Figure 3D). Furthermore, brownish-pink abnormal tissue was observed in the medial-rostral aspect of the joint (Figure 3E). Once the abnormal tissue was identified, a pair of arthroscopic graspers (NanoGrasper, Straight Tip, Arthrex Vet Systems, Naples, FL, USA) was introduced into the joint space rostral to the scope portal in the ventral TMJ compartment, and three biopsies were obtained under NanoScope guidance (Figure 4). No complications occurred during biopsy collection. The sheath was then removed, the joint was irrigated, and a three-layer closure was performed (i.e., muscles and fascia, subcutaneous tissue, and skin). The biopsy samples were diagnostic, and the mass was histopathologically identified as a myxoma vs. a myxosarcoma, confirming the clinical suspicion. The benign vs. malignant versions of this neoplasm are difficult to distinguish histologically; however, after consultation with the pathology team, the clinical behavior, which caused osteolysis and extensive spread, favored a myxosarcoma (Figure 3F). Postoperatively, the dog was placed on amoxicillin/clavulanic acid (15 mg/kg q 12 h for 5 days), maropitant (2 mg/kg q 24 h), and ondansetron (0.3 mg/kg q 8 h). For postoperative pain control, due to prior adverse effects to non-steroidal anti-inflammatories, the dog was placed on galliprant (2 mg/kg q 24 h for 10 days) and amantidine (5 mg/kg q 12 h for 10 days). Follow-up 1 week postoperatively revealed that the patient was comfortable and the owner had no further concerns. The owner then elected to pursue palliative care with the referring veterinarian.

### Case 2

**Figure 5:**
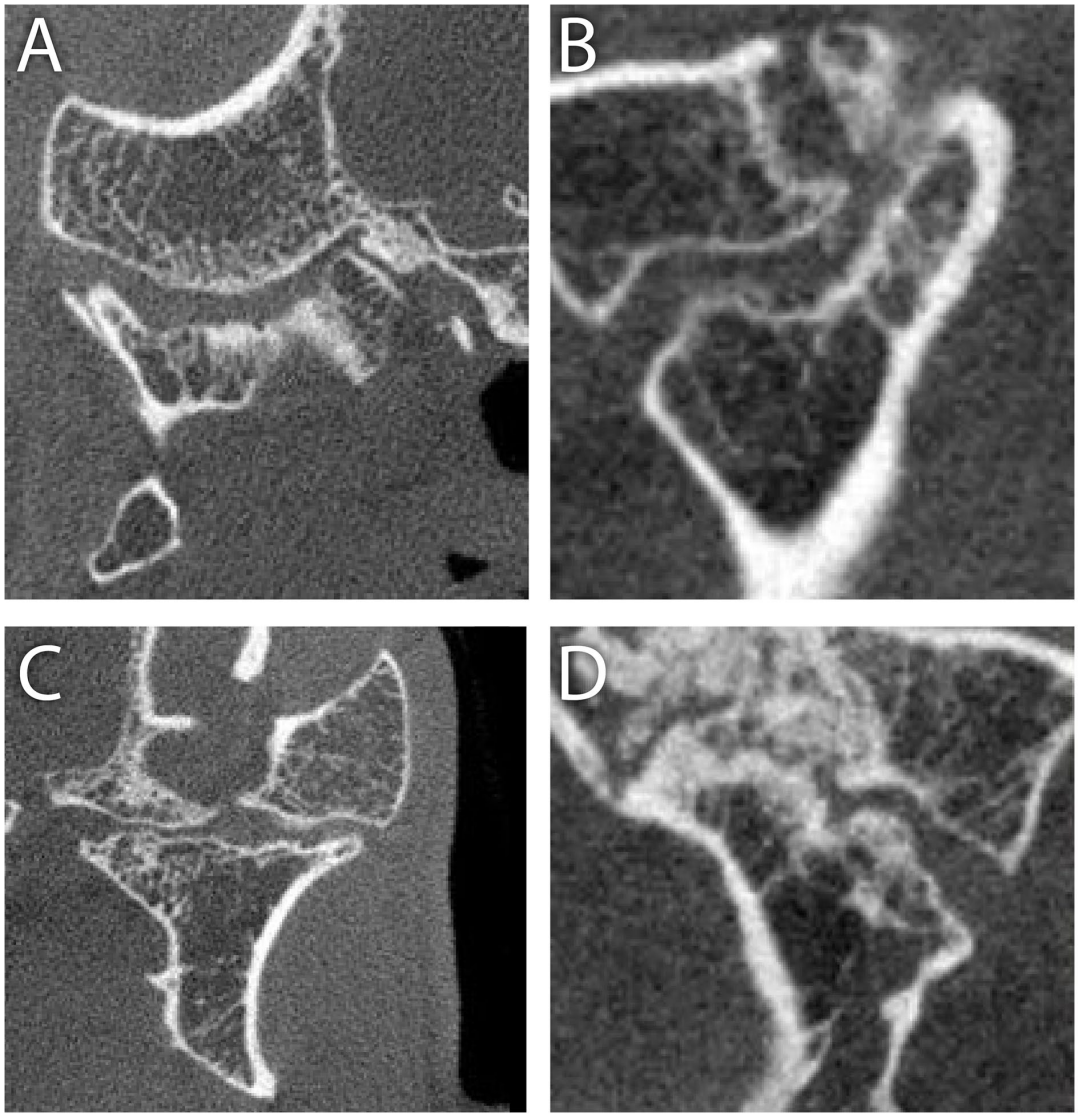
Cone beam CT images in transverse **(A)** and sagittal **(B)** sections at the level of the right TMJ and in transverse **(C)** and sagittal **(D)** sections at the level of the left TMJ in a 5-year-old German shepherd mix, as described in Case 2. The articular surfaces of both the mandibular head and mandibular fossa on both sides exhibit advanced degenerative joint disease, but the joint space is intact with no radiological signs of intra-articular ankylosis.

A 5-year-old, 38.5 kg, German shepherd mix was presented for an inability to open its mouth for approximately 5 years, slight mandibular brachygnathia, and severe generalized periodontal disease. A cone beam CT scan was performed under heavy sedation and revealed severe bilateral intra- and extra-articular TMJ ankylosis, advanced bilateral degenerative joint disease of the TMJ (Figure 5), generalized periodontitis, and slight mandibular brachygnathia. Given the imaging findings and the presence of a visible joint space in the majority of both joints, a bilateral TMJ-sparing arthroplasty was planned. The aim was only to remove the ankylotic tissue and keep the remaining TMJs, which were not directly involved in the ankylosis, in place. This would provide functional support for opening and closing the mouth, in addition to helping maintain function, as opposed to bilateral gap arthroplasty. In addition, after excising the ankylotic tissue and achieving mouth opening, the plan was to investigate the TMJs using arthroscopy and, if needed, remove intra-articular adhesions.

**Figure 6:**
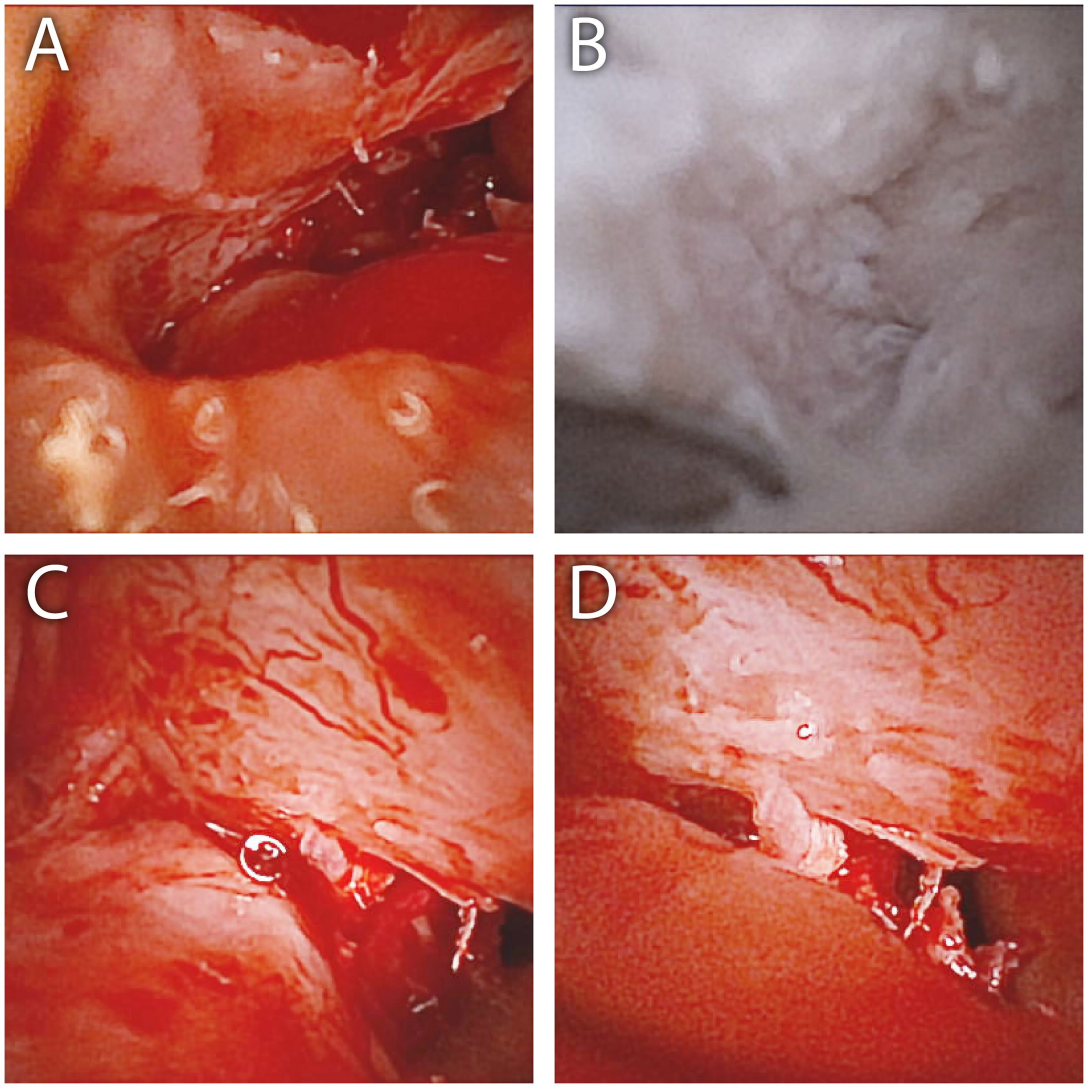
Intra-articular view of the right **(A,B)** and left **(C,D)** TMJs of the dog described in case 2. The joint space of both joints was determined to be intact, and advanced degenerative joint disease was confirmed by increased vascularity, cartilage eburnation, and subchondral bone exposure. Of note, the blood in the joints in **(A,C,D)** is from contamination during the overall surgical procedure and from minor intra-articular bleeding.

Following tracheostomy intubation and positioning the dog in right lateral recumbency, a lateral approach to the TMJ and zygomatic arch was performed as previously described. The first approach was to the right side, as it was deemed to be less severely affected than the left side. Once the ankylotic tissue was removed, the surgical site was temporarily closed, and a similar approach to the left side was made. After removal of the ankylotic tissues of the left side, approximately 60% of normal mouth opening was achieved. At this juncture, the lateral aspect of the left TMJ was exposed, and the NanoNeedle Scope was inserted into the dorsal compartment, as previously described. The articular surfaces of the mandibular fossa and the disc were inspected, revealing advanced degenerative changes to both (Figures 6A,B). In addition, intra-articular adhesions were noted. The sheath was removed and inserted into the ventral compartment as previously described. Similar to the dorsal compartment, severe degenerative changes to the articular surfaces were noted. In addition, fibrous tears were observed in areas of disc attachment. Following thorough lavage of the joint, the surgical site was closed in three layers, and the dog was turned onto its right side again. A similar approach was used, and the right TMJ was inspected with similar findings to those of the left side (Figures 6C,D). At this juncture, the mouth was stretched open, resulting in the detachment of the intra-articular adhesions, allowing for a normal mouth opening.

The dog was hospitalized postoperatively for continued supportive care, which included IV crystalloid therapy, IV antibiotics, IV antiemetics, and pain control (carprofen, 2.2 mg/kg IV, fentanyl, constant rate of infusion (CRI) 3–5 mcg/kg/h, and ketamine, CRI 1–2 mcg/kg/min). The dog was discharged 24 h postoperatively on amoxicillin/clavulanic acid (18 mg/kg q 12 h for 7 days), carprofen (4.4 mg/kg q 24 h for 7 days), gabapentin (10 mg/kg q 8–12 h for 14 days) and a fentanyl patch (150 mcg/h for 3–5 days). Instructions were given to the owner to encourage early and regular mouth-opening to prevent recurrence. At a one-week phone call follow-up, the client reported that the dog was doing overall well and was learning to eat with the mouth open and was drinking with no difficulties. The 2-week recheck revealed that the dog was using its mouth, opening and closing the mouth to 90 mm, did not experience pain or discomfort when its mouth was being opened and closed, and was yawning and barking.

## Discussion

This Methods study is structured based on past research (29) and offers a pragmatic approach to needle arthroscopy of the TMJ in medium-to large-breed dogs, exemplifying its use in clinical practice with two case reports. We demonstrated that arthroscopy of the TMJ in dogs is feasible when using a NanoScope™ arthroscopy system. This solution offers excellent direct visualization of the joint, allowing for precise inspection of both joint compartments and the disc with minimal morbidity. It also enables the capturing of photographs and videos for documentation of TMJ pathological processes. In addition, TMJ arthroscopy allows accurate and precise biopsy collection, particularly in cases where the pathology is located in difficult-to-access areas, such as the medial aspect of the joint.

Arthroscopy has become a common diagnostic and treatment modality for TMJ disorders in humans. Three primary levels of arthroscopic intervention have been described. Level 1 includes diagnostic arthroscopy. Level 2 consists of diagnostic arthroscopy, but also includes an operative aspect. In level 3, arthroscopy is used for advanced interventions, such as repairing TMJ disc damage or performing discopexy (21). In this report, we implemented a pragmatic approach to treating the TMJs in dogs with level 1 interventions; specifically, for direct visualization and inspection, but also for guiding biopsy procedures of abnormal tissues. Clinically, visualizing the pathology of the joint compartments and obtaining diagnostic biopsies (as in Case 1) complemented the information derived from CT scans and resulted in precise clinical-pathological correlation, assisting in the subsequent phase of treatment recommendations and planning.

Given the nature of arthroscopy and, in particular, TMJ interventions using arthroscopy, the skills required for technical proficiency in this technique are fundamentally different, yet complementary, to those required for standard arthrotomy and arthroplasty. While arthrotomy relies on direct visualization of the joint structures and tactile feedback to the surgeon’s hands, arthroscopy uses proprioceptive feedback to the operator’s hands while observing a magnified visual field. The movements while performing arthroscopy in the TMJ are typically small and short (i.e., minute movements) to avoid iatrogenic joint damage. In addition, obtaining tissue specimens is the primary goal of Level 1 operative arthroscopy, and we demonstrated its feasibility when guided by arthroscopy and inserted adjacent to the scope portal. The ability to obtain diagnostic biopsies using direct visualization represents a substantial advantage over arthrocentesis.

In the present report, we started our cadaver-based experimental approach using a needle, followed by a stab incision into the joint, and insertion of the sheath, as is done in other synovial joints (30, 33). However, this approach resulted in disc damage. This is clinically significant because the unguided administration of medication into the TMJs of dogs using a needle has been described in dogs suffering from non-odontogenic orofacial pain associated with temporomandibular joint osteoarthritis and/or dysplasia (34). The data from the present report suggest that unguided needle insertion is likely to result in iatrogenic joint damage, such as articular surface and disc damage. This is not surprising, given the tight and limited joint space in the TMJs, along with the presence of the fibrocartilaginous disc in the middle of the joint. If a closed approach to the TMJs is desired, further research is required to avoid iatrogenic injuries. This may include insufflation of the joint with sterile saline prior to introduction of the obturator (i.e., using a pressurized saline bag) or attempting ventral traction of the mandible to increase the joint space if possible.

TMJ arthroscopy has strategic importance in precision diagnostics of internal joint structures, in addition to being crucial for staging and biopsy sampling, as previously described (21, 25, 27, 35). Degenerative joint disease is the most common TMJ disease in dogs and can be diagnosed with cone-beam computed tomography or conventional CT (7, 11). A recent study evaluated the association between TMJ CBCT and gross and histological changes, such as joint space narrowing, subchondral/cortical bone change (i.e., erosion or lysis), osteophytes, and subchondral bone sclerosis (11). The study demonstrated that the more severe the TMJ degenerative changes, the greater the agreement between CBCT and histology. In addition, TMJ disc perforation was also noted bilaterally in 2 out of 20 dogs (11). TMJ disc perforation is a lesion that can be speculated based on CBCT findings but can only be confirmed via direct visualization through arthroscopic disc removal or necropsy. In addition, TMJ arthroscopy is likely to play an important diagnostic and therapeutic role in the management of septic arthritis of the TMJs of dogs, as it does in humans (35, 36). Dogs with septic TMJ arthritis typically exhibit severe pain and dysfunction, necessitating an urgent and precise diagnosis and targeted therapy (37). The current surgical approach involves obtaining diagnostic samples for culture and sensitivity testing, decompressing the joint and associated abscesses, removing joint debris, and copiously irrigating the joint to reduce inflammation. TMJ arthroscopy is beneficial for all of these surgical objectives (37). Given the range of degenerative and arthritic changes observed in the TMJs of dogs (1, 7), TMJ arthroscopy is well positioned to become an increasingly important diagnostic and therapeutic tool.

## Conclusion

The present methods report, exemplified by two clinical cases, demonstrates the practical and efficient use of needle arthroscopy in the TMJs of dogs. Understanding the fundamental aspects of TMJ arthroscopy utilization for the diagnosis and treatment of TMJ disorders is paramount, especially given the unique anatomical features of TMJs in dogs. A semi-open approach was found to be more practical and less likely to cause iatrogenic damage than a single stab incision. The ability to visualize joint structures in health and disease and perform arthroscopy-guided biopsy is valuable and carries clinically relevant implications. As TMJ arthroscopy in dogs is a developing field, future research should focus on wider clinical experience studies, outcomes, and applications for smaller dogs and cats, in addition to developing an educational curriculum and training for clinicians.

## Data Availability

The raw data supporting the conclusions of this article will be made available by the authors, without undue reservation.
